# Abnormal hemostatic function one year after orthotopic liver transplantation can be fully attributed to endothelial cell activation

**DOI:** 10.12688/f1000research.3980.2

**Published:** 2014-07-30

**Authors:** Freeha Arshad, Jelle Adelmeijer, Hans Blokzijl, Aad van den Berg, Robert Porte, Ton Lisman

**Affiliations:** 1 Surgical Research Laboratory, Department of Surgery, University of Groningen, University Medical Center Groningen, Groningen, 9700 RB, Netherlands; 2 Section of Hepatobiliary Surgery and Liver Transplantation, Department of Surgery, University of Groningen, University Medical Center Groningen, Groningen, 9700 RB, Netherlands; 3 Department of Gastroenterology, University of Groningen, University Medical Center Groningen, Groningen, 9700 RB, Netherlands

## Abstract

**Background:** The long-term risk of thrombotic and vascular complications is elevated in liver transplant recipients compared to the general population. Patients with cirrhosis are in a hypercoagulable status during and directly after orthotopic liver transplantation, but it is unclear whether this hypercoagulability persists over time.

**Aim:** We aimed to investigate the hemostatic status of liver transplant recipients one year after transplantation.

**Methods:** We prospectively collected blood samples of 15 patients with a functioning graft one year after orthotopic liver transplantation and compared the hemostatic status of these patients with that of 30 healthy individuals.

**Results:** Patients one year after liver transplantation had significantly elevated plasma levels of von Willebrand factor (VWF). Thrombin generation, as assessed by the endogenous thrombin potential, was decreased in patients, which was associated with increased plasma levels of the natural anticoagulants antithrombin and tissue factor pathway inhibitor.  Plasma fibrinolytic potential was significantly decreased in patients and correlated inversely with levels of plasminogen activator inhibitor-1.

**Conclusion: **One year after liver transplantation, liver graft recipients have a dysregulated hemostatic system characterised by elevation of plasma levels of endothelial-derived proteins. Increased levels of von Willebrand factor and decreased fibrinolytic potential may (in part) be responsible for the increased risk for vascular disease seen in liver transplant recipients.

## Introduction

Patients with chronic liver disease frequently have major and multiple alterations in their hemostatic system, including a decreased platelet count and decreased plasma levels of pro- and anti-hemostatic proteins produced by the diseased liver ^1^. The decrease in procoagulant proteins is evidenced by prolonged test results of routine coagulation assays such as the prothrombin time (PT) and activated partial thromboplastin time (APTT). Historically, due to an increased bleeding risk during surgery in combination with prolonged conventional coagulation tests and thrombocytopenia, liver disease patients were thought to be in a hypocoagulable state. In recent years it has become increasingly accepted that cirrhosis patients have a rebalanced hemostatic system which is not adequately represented by routine coagulation tests as they are only sensitive for procoagulant proteins and do not take the concomitant decrease in antihemostatic proteins into account ^2, 3^. The rebalanced hemostatic system is more fragile as compared to healthy individuals and may decompensate towards hypo- or hypercoagulability by factors such as renal failure, trauma, infection, and surgery ^1^. Besides bleeding complications, patients with cirrhosis are also at risk for thrombotic complications and this particular clinical scenario has only recently been fully appreciated ^4– 8^.

Patients with cirrhosis who undergo orthotopic liver transplantation (OLT) show a rapid normalisation of coagulation proteins due to the intact synthetic capacity of the transplanted liver. Previous studies performed in our laboratory have shown that hemostatic capacity early after OLT appears adequate, but shows important differences when compared to healthy individuals. At 10 days after OLT, when synthetic function of the liver as assessed by PT and APTT values is adequate, multiple laboratory parameters suggest that the patients are in a hypercoagulable state. Specifically, we have shown an unbalanced von Willebrand factor (VWF)/ADAMTS13 system ^9^ and enhanced thrombin generation ^2^. Also, we have shown a decreased fibrinolytic potential up to five days after surgery ^10^.

Clinically, this hypercoagulable status is evidenced by a profoundly increased risk for thrombotic complications such as hepatic artery thrombosis (HAT). While previously HAT was assumed to be a solely surgical complication, there is emerging evidence for the involvement of the hemostatic system in the development of HAT ^11^. In addition, liver transplant recipients are at increased risk for arterial thrombotic events. The risk for thrombotic complications remains increased months and even years after OLT compared to the general population and a substantial part of morbidity and mortality in liver transplant recipients who survive the first year after transplantation is due to vascular events ^12, 13^. Long-term vascular complications are mainly ascribed to the use of immunosuppressive medication ^12^. Besides the known metabolic risk profile associated with the use of immunosuppressive medication, several *in vitro* studies have provided evidence for a prohemostatic effect of such drugs ^14, 15^.

While there is laboratory evidence for a hypercoagulable state during and directly after OLT, it is unclear whether the hypercoagulability persists and, if so, for how long. To our knowledge there has been no study investigating the hemostatic potential in liver transplant recipients long after a successful transplant. We aimed to investigate the long-term status of the hemostatic system by various assays of hemostatic competence in patients one year after OLT. Understanding the hemostatic state of transplanted patients is essential for clinical practice and for the development of preventive measures for short- and long term vascular complications.

## Methods

### Patients

We designed a prospective cohort study. Fifteen adult patients who visited the outpatient Hepatology Clinic of the University Medical Center Groningen (UMCG) in The Netherlands for their one-year follow-up visit after OLT, and had adequate liver function as assessed by routine laboratory parameters such as aspartate aminotransferase (ASAT) and alanine aminotransferase (ALAT), bilirubin, albumin, and PT, were included in this study. We included 30 healthy volunteers from our laboratory staff (9 males, 21 females – median age (IQR): 31 (25–42)) to establish reference values for the various tests performed in the study. Patients and controls with a history of thrombotic complications, congenital coagulation disorders, active graft rejection, active infection, or who had used anticoagulant drugs in the past 10 days, suffered from disease recurrence, or were pregnant were excluded. A brief questionnaire was used to collect demographic and disease information ( Supplementary File 1).

### Plasma samples

Blood samples were drawn by veni-puncture and collected into vacuum tubes containing 3.8% trisodium citrate as an anticoagulant (Becton Dickinson, Breda, The Netherlands), at a blood to anticoagulant ratio of 9:1. Platelet-poor plasma was prepared by double centrifugation at 2000 *g* and 10.000 *g* respectively for 10 min. Plasma was snap-frozen and stored at -80°C until use.

### Primary hemostasis

Plasma levels of VWF were determined with an in-house enzyme-linked immunosorbent assay (ELISA) using commercially available polyclonal antibodies (A0082 for coating and P0226 for detection, both are rabbit anti-human antibodies, P0226 is a horseradish-peroxidase conjugated version of A0082 (RRID:AB_579516), DAKO, Glostrup, Denmark). A disintegrin and metalloproteinase with a thrombospondin type 1 motif, member 13 (ADAMTS13) activity was measured in plasma which was pretreated for 30 minutes at 37°C with bilirubin oxidase (10U/mL; Sigma-Aldrich, Zwijndrecht, The Netherlands) to avoid interference of bilirubin with the assay. ADAMTS13 activity was assessed using the FRETS-VWF73 assay (Peptanova, Sandhausen, Germany) based on method described by Kokame *et al.* ^16^. The antigen levels of VWF and the activity of ADAMTS13 in pooled normal plasma were set at 100%, and values obtained in test plasmas were expressed as a percentage of pooled normal plasma.

Platelet activation was assessed by measuring plasma levels of soluble P-selectin and platelet factor 4 (PF4) with a commercially available ELISAs (R&D Systems, Abingdon, United Kingdom).

### Thrombin generation

The thrombin generation test was performed using platelet-poor plasma (PPP) with the fluorimetric method described by Hemker, Calibrated Automated Thrombography ^®^ (CAT) ^17^. Coagulation was activated using a commercial trigger composed of recombinant tissue factor (TF) at a concentration of 4 pM and phospholipids at a concentration of 4 μM, in the presence or absence of soluble thrombomodulin (TM) (Thrombinoscope BV, Maastricht, The Netherlands). Thrombin Calibrator (Thrombinoscope BV, Maastricht, The Netherlands) was added to the wells containing plasma to calibrate the thrombin generation curves. A fluorogenic substrate with CaCl _2_ (FluCa-kit, Thrombinoscope BV, Maastricht, The Netherlands) was dispensed in each well to allow a continuous registration of thrombin generation. Fluorescence produced was read every 20 seconds by a fluorometer, Fluoroskan Ascent ^®^ (ThermoFisher Scientific, Helsinki, Finland). All experiments were performed in triplicate.

The endogenous thrombin potential (ETP), peak height, velocity index and lag time were derived from the thrombin generation curves by the Thrombinoscope software.

Level of prothrombin F1+2 fragment in plasma were determined with a commercially available ELISA (Siemens, Breda, The Netherlands) according to the manufacturer’s instructions.

### Routine coagulation laboratory tests

Levels of factor (F) VIII, II, antithrombin (AT) and Protein C were measured on an automated coagulation analyzer (ACL 300 TOP) with reagents and protocols from the manufacturer (Recombiplastin 2G and FII depleted plasma for FII, Hemosil (R) SynthASil and FVIII depleted plasma for FVIII, Liquid Antithrombin reagent for AT, and Hemosil Protein C for Protein C measurements; Instrumentation Laboratory, Breda, the Netherlands).

Plasma levels of Tissue Factor Pathway Inhibitor (TFPI) were determined with an in-house ELISA as previously described ^18^.

### Fibrinolytic potential

Fibrinolytic potential was assessed using a plasma-based clot lysis assay. Lysis of a tissue factor–induced clot by exogenous tissue plasminogen activator (tPA) was studied by monitoring changes in turbidity during clot formation and subsequent lysis as described previously ^19^. In short, 50 μL plasma was pipetted in a 96-well microtiter plate. Subsequently, a mixture containing phospholipid vesicles, tPA, tissue factor, and CaCl _2_, adjusted to a total volume of 50 μL by addition of HEPES (N-2-hydroxytethylpiperazine-N-2-ethanesulfonic acid) buffer (25 mM HEPES, 137 mM NaCl, 3.5 mM KCl, 3 mM CaCl _2_, 0.1% bovine serum albumin, pH 7.4) was added using a multichannel pipette. In a kinetic microplate reader (Versamax, Molecular Devices, Sunnyvale, CA), the optical density at 405 nm was monitored every 20 seconds at 37°C, resulting in a clot-lysis turbidity profile. Clot lysis times were derived from the clot-lysis turbidity profiles using in house-generated software. The clot lysis time was defined as the time from the midpoint of the clear to maximum turbid transition, representing clot formation, to the midpoint of the maximum turbid to clear transition, representing the lysis of the clot.

Plasma levels of plasminogen activator inhibitor-1 (PAI-1) levels were determined with a commercially available ELISA (Sekisui, Stamford, USA).

### Statistical analyses

Data are expressed as means (with standard deviations (SDs)), medians (with interquartile ranges), or numbers (with percentages) as appropriate. Means of two groups were compared by Student’s t-test or Mann-Whitney U test as appropriate. Spearman’s correlation coefficient was used to assess correlation between continuous variables. P values of 0.05 or less were considered statistically significant. GraphPad Prism (San Diego, USA) and IBM SPSS Statistics 20 (New York, USA) were used for analyses.

### Ethics statement

Written informed consent was obtained from every participant in this study. The study was approved by the local Medical Ethics Committee from the University Medical Center of Groningen (protocol number 2012.098). Study procedures were in accordance with the Helsinki Declaration of 1975.

## Results

### Patient characteristics

All of the patients included in this study underwent OLT between 2011 and 2012. All patients received a full-size graft. None of the patients suffered from thrombosis prior to OLT or had postoperative thrombotic complications within the first year. Five patients suffered from diabetes mellitus at time of the blood draw, and four of these were insulin-dependent. Two of these patients had developed diabetes after OLT. There were five patients that were on platelet aggregation inhibitors (calcium carbasalate or aspirin) at the time of the blood draw. Two of these patients had coronary disease for which they had undergone coronary interventions prior to OLT. One patient had left ventricular hypertrophy and one patient had paroxysmal atrial fibrillation. The fifth patient appeared to have fragile arteries at the anastomotic site during OLT for which post-operative aspirin was started. Two patients suffered from hypertension, and two patients smoked cigarettes. Patient and background characteristics are presented in Table 1.

**Table 1.** Patient characteristics.

Mean age (years) (± SD)		50.0 (± 1.9)
Male/female ratio		11/4
Mean BMI (± SD)		26.0 (± 2.9)
Etiology of liver disease (no of patients)	Biliary cirrhosis	5
	Alcoholic cirrhosis	2
	Viral cirrhosis	3
	Acute liver failure	1
	Familial amyloidotic polyneuropathy	1
	Morbus Wilson	1
	Alcoholic cirrhosis and NASH	1
	NASH	1
Piggyback/conventional implantation		12/3
Donor type	Heartbeating	10
	Non-heartbeating	4
	Domino	1
Immunosuppressive regimen (no of patients)	Calcineurin inhibitor	2
	Calcineurin inhibitor + steroid	1
	Calcineurin inhibitor + steroid + purine antagonist	10
	Calcineurin inhibitor + purine antagonist	2
Other medication (no of patients)	Aspirin	5
	Insulin	4
	Metformin	2
	Calcium antagonist	4
	ACE-inhibitors	4
	Betablocker	1
	Diuretic	1
	Proton pump inhibitor	13
Laboratory assessment (medians and IQR)	Hemoglobin (mmol/L)	8.6 (8.2–9.0)
	Platelets (×10 ^9^/L)	160 (136–192)
	Total bilirubin (µmol/L)	9.0 (6.0–11.0)
	ASAT (U/L)	26 (18–3)
	ALAT (U/L)	25 (20–38)
	Albumin (g/L)	45 (44–47)
	INR	1.1 (1.0–1.1)
	Creatinine (µmol/L)	77 (72–107)

*To convert values for hemoglobin to g/dl, multiply by 1.650. To convert values for bilirubin to mg/dl divide by 88.4.

### A dysbalanced VWF/ADAMTS13 ratio in liver transplant recipients

**Figure 1.**
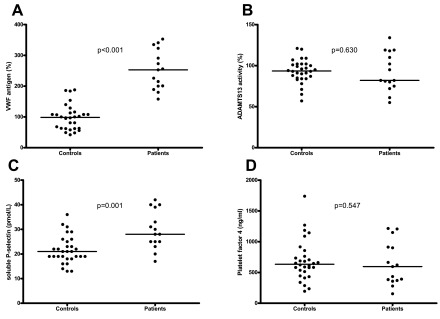
VWF and Platelet parameters in healthy controls and patients. **A**. Plasma levels of von Willebrand factor (VWF) in patients and healthy controls. **B**. ADAMTS13 activity in plasma from patients and healthy controls. **C**. Plasma levels of soluble P-selectin in patients and controls. **D**. Plasma levels of Platelet Factor 4 in patients and controls. Horizontal bars indicate medians.

Patients had significantly higher plasma levels of the platelet-adhesive protein VWF compared to healthy controls (253% (200–323) (median (IQR)) vs. 99% (63–114), respectively, Figure 1A). The activity of ADAMTS13, the VWF-cleaving protease was comparable between patients and controls (82% (75–118) vs. 94% (85–102) respectively, Figure 1B). Plasma levels of sP-selectin were significantly elevated in patients compared to controls (28.0 pmol/L (25.0–39.0) vs. 21.0 pmol/L (18.8–25.3) respectively, Figure 1C). Levels of sP-selectin were similar in patients that were on calcium carbasalate or ascal compared to those who were not (33.0 pmol/L (20.0–41.0) vs. 28.0 pmol/L (25.0–33.0) respectively; p=0.68). However, levels of PF4 were similar among patients and controls (595 ng/ml (369–912) vs. 634 ng/ml (496–786) respectively; Figure 1D).

### Decreased *in vitro* thrombin generation is associated with elevated plasma levels of TFPI and AT, but not with differences in *in vivo* thrombin generation in liver transplant recipients

**Figure 2.**
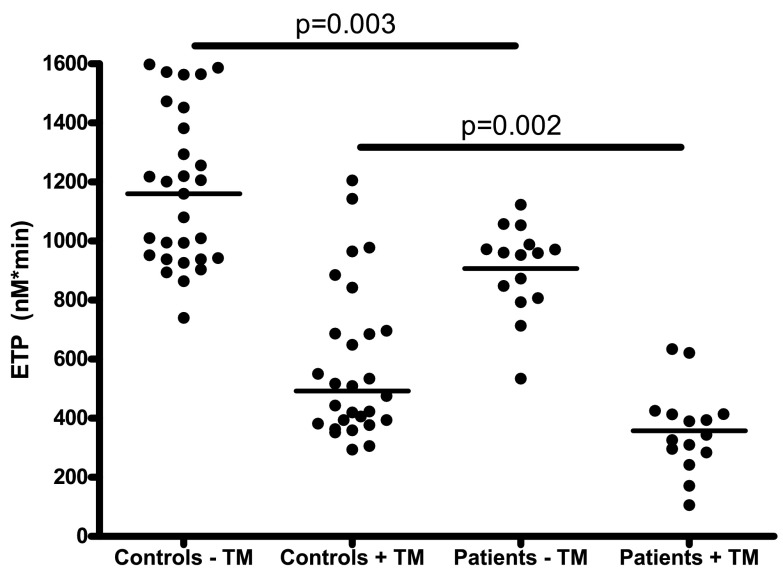
Endogenous Thrombin Potential (ETP) in plasma from patients and healthy controls in absence and presence of thrombomodulin (TM). Horizontal bars indicate medians.

Thrombin generation assays showed that patients had a decreased procoagulant capacity, both in presence and absence of thrombomodulin ( Figure 2). Specifically, patients had a decreased ETP compared to controls, both in presence and absence of thrombomodulin (344 nM IIa×min (284–414) vs. 492 nM IIa×min (385–693) respectively in presence of thrombomodulin). Patients also had a decreased peak height and velocity index, and a prolonged lagtime compared to controls ( Table 2).

**Table 2.** Parameters derived from thrombin generation curves generated in absence and presence of TM. Data are presented as medians with interquartile range.

	Patients	Controls	p-value
ETP ratio	0.4 (0.3–0.4)	0.5 (0.3–0.8)	0.037
Velocity index TM- (nM IIa/min)	55.0 (43.0–67.0)	73.0 (56.0–134.0)	0.004
Velocity index TM+ (nM IIa/min)	43.0 (34.0–54.0)	58.5 (46.8–93.8)	0.002
Peak TM- (nM IIa)	172.0 (162.0–196.0)	190.0 (182.0–306.0)	0.004
Peak TM+ (nM IIa)	98.0 (78.0–98.0)	134.0 (100.0–187.0)	0.003
Lag time TM- (min)	2.7 (2.3–2.7)	2.0 (1.7–2.0)	<0.001
Lag time TM+ (min)	2.3 (2.0–2.5)	1.7 (1.7–2.0)	<0.001

The ETP ratio, an index of the anticoagulant capacity of the protein C system defined as the ratio of the ETP with-to-without TM, was significantly lower in patients compared to controls ( Table 2).

**Figure 3.**
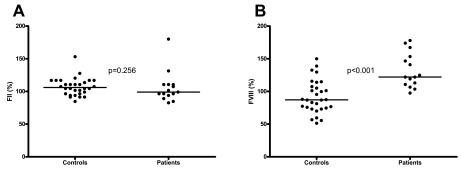
Coagulation factor levels in patients and healthy controls. **A**. Plasma levels of factor II in patients and healthy controls. **B**. Factor VIII plasma levels in patients and healthy controls. Horizontal bars indicate medians.

Plasma levels of FII were similar in patients and controls (99% (94–111) vs. 106% (96–117) respectively, Figure 3A). Levels of FVIII on the other hand were significantly higher in patients compared to controls (122% (111–153) vs. 87% (74–109) respectively, Figure 3B).

**Figure 4.**
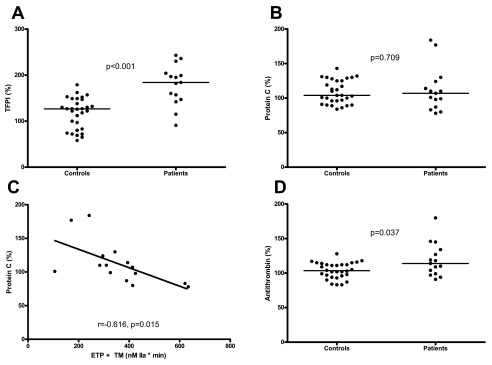
Coagulation factor levels in patients and healthy controls. **A**. Plasma levels of TFPI in patients and healthy controls. **B**. Protein C levels in plasma from patients and healthy controls. **C**. Correlation between the Endogenous Thrombin Potential (ETP) measured in the presence of thrombomodulin (TM) and plasma levels of protein C in patients. **D**. Antithrombin (AT) levels in plasma from patients and healthy controls. Horizontal bars indicate medians.

Plasma levels of TFPI were significantly higher in patients compared to controls (184% (147–204) vs. 127% (82–148) respectively, Figure 4A). There was no difference in protein C levels between the groups (107% (87–124) vs. 104% (95–126) respectively, Figure 4B). In the patient group protein C correlated inversely with ETP in presence of TM ( Figure 4C). Levels of AT were slightly, but significantly higher in patients compared to controls (114% (99–134) vs. 104% (97–113) respectively, Figure 4D).

**Figure 5.**
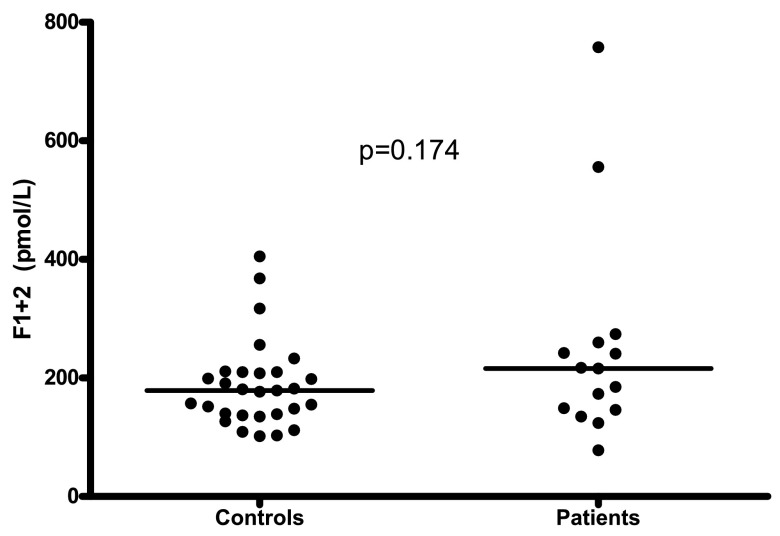
Plasma levels of prothrombin fragment 1+2 in patients and healthy controls. Horizontal bars indicate medians.

Plasma levels of prothrombin fragment 1+2, an indicator of *in vivo* thrombin generation, were similar between patients and controls (216 pmol/L (146–260) vs. 178 pmol/L (136–210) respectively, Figure 5).

### Decreased plasma fibrinolytic potential associated with elevated plasma levels of PAI-1 in liver transplant recipients

**Figure 6.**
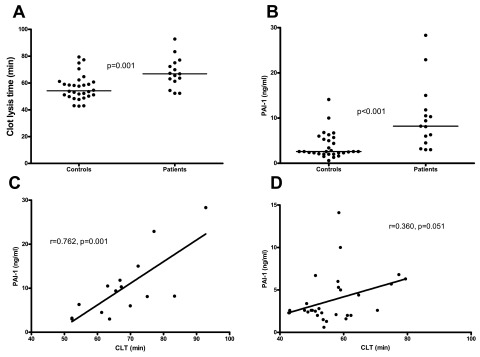
Fibrinolytic status in patients and healthy controls. **A**. Clot lysis time assessed in plasma from patients and healthy controls. **B**. Plasma levels of PAI-1in patients and healthy controls. **C**. Correlation between clot lysis times and PAI-1 plasma levels in patients and **D**. controls. Horizontal bars in panels A and B indicate medians.

Clot lysis times were significantly prolonged in patients compared to controls (66.8 min (61.3–75.1) vs. 54.2 min (50.1–60.8) respectively Figure 6A and B). Plasma levels of PAI-1 were significantly higher in patients compared to controls (8.2 ng/ml (4.5–11.8) vs. 2.1 ng/ml (2.6–5.4) respectively) and correlated with clot lysis time ( Figure 6C and D).

Database containing demographic data of each patient and laboratory data of each patient and control This file contains raw data of all laboratory measurements presented in the paper. In addition, the file contains raw demographic data of the patients as summarized in the paper in Table 1.

## Discussion

The main finding of this study is that one year after OLT patients with a well-functioning graft are in a hypercoagulable state. This hypercoagulable state is caused by elevated plasma levels of VWF (resulting in a hyperactive primary hemostatic system), and a hypofibrinolytic state due to elevated plasma levels of PAI-1. Nevertheless, thrombin generation was decreased in patients one year after OLT, which was related to increased levels of TFPI and AT, which were previously shown to be key determinants of thrombin generation in healthy individuals ^20^. This decreased thrombin generation may, in part, compensate for the hypercoagulable changes. High VWF and decreased plasma fibrinolytic potential, however, are established risk factors for venous and arterial thrombosis, and we therefore speculate that the unbalanced hemostasis in patients one year after OLT may contribute to their increased risk for thrombotic events. The observed increased levels of VWF, sP-selectin, FVIII, TFPI, and PAI-1, in patients one year after OLT, are likely the result of chronic endothelial injury. All of these coagulation proteins are produced by endothelial cells. It has been demonstrated that the use of immunosuppressive drugs leads to endothelial cell activation and release of VWF ^14, 21– 26^. Also, an inhibitory effect of immunosuppressive medication on fibrinolysis has been demonstrated, as evidenced by elevated levels of PAI-1 ^26– 28^.

It thus appears plausible that the elevation in levels of VWF, sP-selectin, FVIII, TFPI, and PAI-1, which explain the majority of the hemostatic unbalance at one year after OLT, is related to immunosuppression. Nevertheless, other causes for endothelial activation such as ( *de novo*) diabetes, smoking, and infection or a proinflammatory status may also contribute to endothelial cell activation. Of note, none of the other drugs used by some of the patients, such as proton pump inhibitors, blood-glucose lowering medication or calcium antagonists are known to cause endothelial cell activation.

Previously we have summarized clinical and laboratory evidence for hypercoagulability as a contributor to thrombotic complications after liver transplantation ^11^. Several of the hemostatic abnormalities that we have described in the present study have been linked to clinical thrombotic events in non-transplant patients and, therefore, are in line with our previously formulated hypotheses. Increased levels of VWF and FVIII have been (independently and in combination) described as a risk factor for venous thromboembolism but also for thromboembolic cardio- and cerebrovascular disease and mortality in several studies ^29– 32^. Hypofibrinolysis, as defined by prolonged plasma-based clot lysis times or by increased plasma levels of PAI-1 have been associated with a risk for venous thrombosis and thromboembolic cardiovascular disease ^33– 37^.

With increasing long term survival after OLT, thromboembolic cardio- and cerebrovascular disease has become an increasingly threatening factor for OLT recipients, warranting preventative measures ^11^. While there are no laboratory studies investigating the hemostatic status of patients 5–10 years after their OLT, it is plausible that the hemostatic abnormalities induced by immunosuppressive drugs described in this study persist or even aggravate over time. The findings of this study may point to the necessity of antihemostatic treatment to prevent cardiovascular disease after OLT. While both sP-selectin and PF4 are considered markers of platelet activation, only PF4 is solely released by activated platelets. P-selectin is present in both endothelial cells as well as platelets, and a soluble fragment is released upon activation of these cells. The fact that only levels of sP-selectin were elevated in liver transplant recipients while levels of PF4 were similar to controls doesn’t indicate an increased platelet activity but rather increased endothelial cell activation. However, the elevated levels of VWF may lead thromboembolic events, which may be prevented by anti-platelet therapy. It has been demonstrated in a single retrospective study that long-term administration of aspirin lowers the incidence of HAT after OLT without increasing bleeding events ^38^. Platelet inhibition after OLT by aspirin might not only decrease the risk for HAT but also the risk for thromboembolic cardiovascular disease similar to that in the general population, although this has not been assessed in clinical studies.

The results of our study suggest that the hemostatic imbalance of liver transplant recipients is not due to transplant-related effects, but to the endothelial activating properties of immunosuppression, perhaps in combination with endothelial activation associated with comorbidities such as diabetes and smoking. Our results therefore may be likely extended to other forms of solid organ transplantation. Indeed, kidney transplant recipients are also at risk for thrombotic events, which may also in part be related to dysregulated hemostasis ^39– 42^. Although antihemostatic therapy may be beneficial, partial or complete withdrawal of immune suppression may contribute to avoiding post-transplant thrombotic complications. In addition, adjustment of lifestyle (e.g., cessation of smoking), and optimal control of diabetes and hypertension may contribute to a decreased risk of post-transplant thrombosis.

In conclusion, one year after liver transplantation liver transplant recipients display dysregulated hemostasis which appears to be related to endothelial activation. Whereas elevated levels of VWF and decreased fibrinolytic capacity may be related to thrombotic complications in liver transplant recipients, this risk may be attenuated in part by decreased thrombin generating capacity.

## Data availability

*figshare*: Database containing demographic data of each patient and laboratory data of each patient and control. Doi: 10.6084/m9.figshare.1002065 ^43^

## Informed consent

Written informed consent was obtained from participant in this study.
