# Supplementary material for: Abnormal hemostatic function one year after orthotopic liver transplantation can be fully attributed to endothelial cell activation
Source: F1000Res. 2014 Jul 30;3:103. Originally published 2014 May 9. [Version 2] doi: 10.12688/f1000research.3980.2 (PMC4176425; doi:10.12688/f1000research.3980.2)
Supplement: Peer review history

# Peer review: Referee response for version 2

**DOI:** 10.5256/f1000research.5207.r5648

**Published:** 2014-08-14

**Authors:** Alex Gatt

**Affiliations:** 1 Haematology Department, Mater Dei Hospital, Msida, Malta

No more comments. Thanks

I have read this submission. I believe that I have an appropriate level of expertise to confirm that it is of an acceptable scientific standard.

# Peer review: Referee response for version 2

**DOI:** 10.5256/f1000research.5207.r5649

**Published:** 2014-08-11

**Authors:** Annabel Blasi

**Affiliations:** 1 Hospital Clinic de Barcelona, Barcelona, Spain

It´s OK! I've no more comments.

I have read this submission. I believe that I have an appropriate level of expertise to confirm that it is of an acceptable scientific standard.

# Peer review: Referee response for version 1

**DOI:** 10.5256/f1000research.4265.r5188

**Published:** 2014-07-04

**Authors:** Alex Gatt

**Affiliations:** 1 Haematology Department, Mater Dei Hospital, Msida, Malta

Arshad *et al.* present an interesting study on the haemostatic changes seen 1 year after liver transplantation. This is of particular interest to hepatologists, liver surgeons and haemostasiologists alike. The Groningen group clearly show that these patients, even 1 year after transplantation, still have high FVIII, and VWF. However they do recover their anticoagulant proteins, leading to a brisk reduction of thrombin generation upon the addition of thrombomodulin.

This is a proof of concept study due to the small number of patients. There is a major confounder, as the authors themselves acknowledge in their introduction, which is the chronic immunosuppression which could be leading this 'endothelial activation', but nevertheless whatever the cause it looks likely that this is present and might play a part in the post transplant thrombotic events in these patients. 

- Ideally, the authors should specify what Protein C and AT assays they used, i.e. are they activity assays?
- Another confounder is that some patients were on antiplatelet agents and other were not.
- It is not clear why the patients still had lower thrombin generation without thrombomodulin. This could be related to the concentration of TF used in the assay.
- Were the controls age and sex matched?
- Finally, despite the fact that VWF, FVIII and PAI-1 are still elevated 1 year after transplantation, looking at the TG data, the patients seem to be hypocoagulable with lower Velocity Index, prolonged LT and lower ETP. There is also a seemingly more profound effect of the natural anticoagulants upon addition of TM. So is this still a case of balanced haemostasis?

I have read this submission. I believe that I have an appropriate level of expertise to confirm that it is of an acceptable scientific standard.

# Response

**Published:** 2014-07-22

**Authors:** Ton Lisman

**Affiliations:** Faculty of Medical Sciences, Surgery, University Medical Center Groningen, Netherlands

We appreciate the interest of the reviewer in our study and thank him for his constructive comments.

1. The Protein C and AT assays are indeed activity assays using chromogenic substrates. As indicated in the manuscript, these assays were purchased from Instrumentation Laboratory. For a detailed description of the assays see: http://benelux.instrumentationlaboratory.com/products-services/hemostasis-diagnostics/reagents/thrombophilia.aspx.
2. Although we agree that the use of antiplatelet agents may be a confounder (although antiplatelet agents to our knowledge have not been shown to alter the outcome of most of the assays we have used), we would like to stress that the clinical reality is that liver transplant recipients are frequently prescribed multiple drugs other than immunosuppressants. We were interested to find out whether coagulation abnormalities may contribute to the increased thrombotic risk after liver transplantation. Thus, while acknowledging the fact that the causes of the hemostatic changes observed in liver transplant recipients are likely multicausal (and not fully elucidated by our study), our data show evidence for a hypercoagulable profile in patients one year after transplantation.
3. The reduced thrombin generation in the absence of thrombomodulin is likely due to the elevated levels of TFPI and AT, which have been shown to be key determinants of thrombin generation ( J Thromb Haemost. 2008 Jan;6(1):125-31). This reference has been added to the manuscript.
4. Controls were not age and sex matched. We included controls to establish reference values for the various assays performed in the study, similar to how reference values in routine diagnostic laboratories are established. While acknowledging that not all measured variables are constant with age, the differences observed in the endothelial-derived proteins are substantial and not explained by minor changes caused by age. We have altered the text in the materials and methods section to indicate that controls were included only to establish reference ranges, and included age and sex of the controls.
5. We agree that we find evidence of both hyper- and hypocoagulable changes in the liver transplant recipients. Our conclusion thus is not that the hemostatic system is in balance, as suggested by the reviewer. Rather, we conclude there is dysregulated hemostasis. The final sentence of the conclusion sums up how we interpret our findings of the study: “Whereas elevated levels of VWF and decreased fibrinolytic capacity may be related to thrombotic complications in liver transplant recipients, this risk may be attenuated in part by decreased thrombin generating capacity.”

# Peer review: Referee response for version 1

**DOI:** 10.5256/f1000research.4265.r5186

**Published:** 2014-06-23

**Authors:** Annabel Blasi

**Affiliations:** 1 Hospital Clinic de Barcelona, Barcelona, Spain

In this study, the authors investigated the hemostatic status of liver transplant recipients one year after transplantation, finding a prothrombotic profile based on activated endothelial markers coupled to a hypofibrinolytic state. It point out a very relevant clinical problem as it is the thrombotic complications in the post transplant period. The aim and methods are well defined and well performed, however I would like the authors address some aspects about the study population:

The study population shows several factors which have clearly impact on the endothelial function, apart from immunosuppressive therapy. In order to obtain more information from the data, I suggest that the authors discuss the differences, if any, about the more relevant results: VW, VIII, sP-selectin, TFPI and PAI-1 according to the following sub-groups of patients:

1. Diabetes
2. Cardiovacular disease
3. Etiology of liver disease - Given that a hypercoagulable/hypofibrinolytic state has been suggested in several of the reported etiologies in the study group (biliary cirrhoris, NASH, familial amyloidotic polyneuropathy) those reversibilities after liver transplantation have not been proven.
4. Immunosuppressive regiment.

To discard that some of the aforementioned conditions confers a risk extra for thrombosis.

Otherwise, patients had a decreased ETP compared to controls, both in presence and absence of thrombomodulin. The ETP ratio, an index of the anticoagulant capacity of the protein C system defined as the ratio of the ETP with-to-without thrombomodulin was significantly lower in patients compared to controls but interestingly, there was no difference in protein C levels between both groups.

Five patients were on platelet aggregation inhibitors. Levels of sP-selectin were similar in patients that were on aspirin compared to those who were not. Could the authors make some comment about this? Aspirin resistance in this population could be suggested?

Although the size and the characteristics of the study population entail some limitations, it is a good starting point (never addressed before), for further studies with a larger sample.

I have read this submission. I believe that I have an appropriate level of expertise to confirm that it is of an acceptable scientific standard.

# Response

**Published:** 2014-07-22

**Authors:** Ton Lisman

**Affiliations:** Faculty of Medical Sciences, Surgery, University Medical Center Groningen, Netherlands

We appreciate the interest of the reviewer in our study and thank her for her constructive comments.

1. The suggestion to look at subgroups is appreciated, and we agree that comorbidities such as diabetes and cardiovascular disease may in itself have an effect on levels of endothelial-derived proteins. In addition, we agree that the etiology of liver disease prior to transplant may be of interest (although the patients were only included when they did not have evidence of disease recurrence), and that the different immunosuppressive regimens may have different effects on levels of endothelial-derived proteins. However, we feel that the small size of our study doesn’t allow for a meaningful subgroup analysis. The normal ranges of the proteins and tests studied are wide already in the general population (also evidenced by the ranges observed in our 30 healthy controls), which indicates that subgroups should be reasonably sized to allow a meaningful subgroup analysis.
2. We agree that the similar P-selectin levels in the patients that were on aspirin  therapy may indicate aspirin resistance, although we did not assess platelet function directly and the group of patients on aspirin was small. It has to be noted, however, that the circulating P-selectin is in part also released from endothelial cells, and this process is not expected to be influenced by aspirin. Nevertheless, liver transplant recipients often have multiple factors (diabetes, smoking, hyperlipidemia that may increase the risk for aspirin resistance. To our knowledge there are no published data on the frequency of aspirin resistance in liver transplant recipients or patients with liver disease and such studies will be required to optimize antihemostatic therapy in these patients.

# Peer review: Referee response for version 1

**DOI:** 10.5256/f1000research.4265.r4734

**Published:** 2014-06-05

**Authors:** Hugo ten Cate

**Affiliations:** 1 Center for Thrombosis and Haemostasis, Johannes Gutenberg University Mainz, Mainz, Germany

Lisman and colleagues follow up on a number of prior studies of hemostatic and coagulation activity status in patients with liver cirrhosis. In this small study they document the hemostatic phenotype in plasma from 15 patients that had underwent orthotopic liver transplantation (OLT) for liver cirrhosis of diverse nature. In the cross sectional study they compare with healthy persons (of comparable age and sex?).

The main finding is that as compared to healthy persons the 15 patients had elevated plasma levels of von Willebrand factor (vWF), plasminogen activator inhibitor-1 (PAI-1), FVIII and soluble P selection, suggestive of an activated endothelium (and/or activated hemostasis) and an attenuated fibrinolytic system, as shown with clot lysis methodology. Interestingly, thrombin generation parameters were either not different (F1+2) or lowered in case of the potential to form thrombin (measured by Calibrated Automated Thrombinography).

The authors conclude that there is evidence of a hypercoagulable state at one year after OLT in these patients, given the above changes, which are suggestive of endothelial activation or damage. A major contributing factor in this process may be the use of immunosuppresive agents. 

In addition they document a hypofibrinolytic state.

Minor comments:

Obviously, this study cannot give insight in the origin of the found abnormalities and it is impossible to dissect effects of immunosuppression from those of underlying morbidity; there are several risk factors for CV disease present in this population that may play a role in endothelial activation. 

A question would be whether any of these patients had been characterised prior to OLT, since it is known that many hemostatic abnormalities are present in association with liver cirrhosis, as the authors also have documented in previous publications. For instance the high level of vWF must (likely) have been present before surgery so that it remains a question how much of the present findings are due to immunosuppression, or to prior comorbidity? 

Also, the comparison with healthy persons from the lab (age? sex?) may suggest that differences in markers are substantial while in part these may be due to young age or healthy status in controls, so the differences attributable to OLT and/or immunosuppression may be smaller when compared to  a comparable group of controls, rather than healthy laboratory subjects.

Interestingly, indeed the hemostatic activation may be counterbalanced by a lowered thrombin generation activity, at least by CAT, so that the net hyper coagulability is somewhat blunted. Given somewhat elevated levels of AT and TFPI, these may explain the lowered ETP and peak levels, but there is no direct evidence that these are the major modifiers in this case.  

In summary, this is a small but interesting study, suggesting a prothrombotic plasma phenotype based on activated endothelial markers coupled to a hypofibrinolytic state, related to elevated PAI-1. Prospective studies of the actual changes pre and post OLT may be needed to get a better idea of the contribution of comorbidity to these changes, preferably as compared to individuals of similar sex and age. The relevance of the observed changes for predicting the risk of thrombosis in these vulnerable patients is worth pursuing.

I have read this submission. I believe that I have an appropriate level of expertise to confirm that it is of an acceptable scientific standard.

# Response

**Published:** 2014-07-22

**Authors:** Ton Lisman

**Affiliations:** Faculty of Medical Sciences, Surgery, University Medical Center Groningen, Netherlands

We appreciate the interest of the reviewer in our study and thank him for his constructive comments.

 

1. We agree that our study is not able to differentiate between the effects of immunosuppression and the effects of comorbidities and smoking on levels of endothelial-derived proteins. We have adjusted the conclusion section to indicate that both immunosuppression and comorbidities may be responsible for the increased levels of endothelial-derived proteins in transplant recipients.
2. The patients were not characterized prior to liver transplantation. Indeed, patients with liver disease have multiple alterations in their hemostatic system which are attributed to defective hepatic synthesis, defective hepatic clearance, consumption, and endothelial activation (which in patients with cirrhosis is strongly related to portal hypertension). Many of the disease-related changes in the hemostatic system are likely no longer present at one year after transplantation in patients with a well-functioning graft. Defects in synthesis and clearance are no longer present in patients with a functioning graft, and also portal hypertension will be no longer present. We thus expect the hemostatic changes observed in the present study not to be related to the pre-transplant liver disease, in particular since patients that had disease recurrence were not included in our study. 
3. As indicated in our response to the comments of Dr. Gatt, our controls were purposely not matched for age and sex (now indicated in the materials and methods section), and were indeed much younger than the age of our patients. We agree that some of the markers are age-dependent, but the increases observed in endothelial-derived proteins are much larger than expected based on age differences only. We nevertheless agree with the reviewer that the differences may be slightly overestimated as a result of the age difference.
4. Although we agree that we did not directly show that the decreased thrombin generation in our patients was related to increased TFPI and AT levels, there is evidence that these 2 proteins are important modifiers of the thrombin generation test. We have added a reference to a paper showing the importance of TFPI and AT in determining thrombin generation.
